# Expanding the indications for measurement of objective functional impairment in spine surgery: A pilot study of four patients with diseases affecting the spinal cord

**DOI:** 10.1016/j.bas.2022.100915

**Published:** 2022-07-20

**Authors:** Gregor Fischer, Vincens Kälin, Oliver P. Gautschi, Oliver Bozinov, Martin N. Stienen

**Affiliations:** a Department of Neurosurgery & Spine Center of Eastern Switzerland, Cantonal Hospital of St.Gallen, St.Gallen, Switzerland; b Neuro- and Spine Center, Hirslanden Clinic St. Anna, Lucerne, Switzerland

**Keywords:** Objective outcome measure, Objective functional impairment, Spinal cord disease, Myelopathy, Spinal cord tumor, Spinal cord herniation

## Abstract

• The TUG test and the 6WT are feasible to apply in ambulatory patients with spinal cord affection.
• The objective measurement of functional impairment resembled the functional status of four patients in this pilot study.
• The TUG test and the 6WT may detect and quantify functional decline and/or improvement resulting from spinal cord affection.
• Modern smartphone app-based technology may help to accurately quantify impairment, which is key for decision-making and evaluating the efficacy of interventions.

## Abbreviations and acronyms:

6WT: 6-minute walking test
BMRC: British Medical Research Council
GPS: Global positioning system
MCID: minimum clinically important difference
MEP: motor-evoked potentials
mJOA: Modified Japanese Orthopedic Association
MRI: Magnetic resonance imaging
OFI: objective functional impairment
PROMs: patient-reported outcome measures
SSEP: somatosensory evoked potentials
SDs: standard deviations
TUG: Timed-Up-and-Go
WHO: World Health Organisation

## 1 Introduction

Determining functional impairment in spine surgery is key for therapeutic decision-making and to critically evaluate the efficacy of therapeutic interventions. While this is often facilitated by purely subjective questionnaire-based patient-reported outcome measures (PROMs) today, objective methods are on the rise to complement the comprehensive patient evaluation (Maldaner et al., 2019; Stienen et al., 2019a; Bostelmann et al., 2016).

The broad availability of smartphones, smartwatches and other wearable devices facilitates the collection of “digital biomarkers” that accurately represent the degree of objective functional impairment (OFI) in a patient (Stienen et al., 2019a, 2020; Harrop, 2020; Smeets et al., 2006; Smuck et al., 2018; Yamaguchi, 2020; Akeret et al., 2018). Two of the most commonly used measures of OFI for patients with degenerative diseases of the lumbar spine are the Timed-Up-and-Go (TUG) test and the 6-min walking test (6WT) (Stienen et al., 2019a). Both tests have been thoroughly evaluated, have favorable test qualities, both normal population data and free smartphone applications for the easy determination of OFI severity are available and patient acceptance is high (Stienen et al., 2019a; Gautschi et al., 2016, 2017; Hartmann et al., 2016; Joswig et al., 2017; Maldaner et al., 2020a; Maldaner and Stienen, 2020; Sosnova et al., 2020). It is not known, however, whether these tests represent OFI in patients with diseases that affect the spinal cord.

The aim of this study was to evaluate the feasibility of both the TUG test and the 6WT as objective, smartphone app-based outcome measures for diseases that affect the spinal cord, including intradural/-intramedullary tumors, spinal cord tethering/spinal adhesive arachnopathies and spinal cord herniation.

## 2 Methods

### 2.1 Patient identification

For this pilot study and technical report, we included four ambulatory patients who presented in the last author’s outpatient clinics or while on-call with diseases affecting the spinal cord between 06/2021 and 08/2021. We deliberately chose various pathologies (intramedullary tumor, extramedullary tumor, spinal cord herniation, spinal cord tethering/arachnopathy) to evaluate the feasibility of applying objective outcome measures in different settings. We did not consider patients who were not ambulatory due to severe spinal cord dysfunction or traumatic conditions with gross instability (where objective functional testing would be considered unsafe).

### 2.2 Patient evaluation

All patients were evaluated pre- and until six weeks postoperatively by standard neurological examination. In addition, one or both tests to determine OFI (TUG test and/or 6WT) were applied, using the free smartphone apps for calculation of age- and sex-specific Z- or T-scores. More information on the apps can be found in the appendix of this article.

For the TUG test, patients were instructed to sit on an armchair with their arms resting on the armrests while leaning back (Gautschi et al., 2016; Maldaner and Stienen, 2020). On command of the examiner, the time was recorded (in seconds (s)) as patients stood up and walked as fast as possible (without running) to a marked line at 3 ​m distance from the chair, where they made a 180° turn, returned and sat down as quickly as possible. Timing was stopped when the participant sat down again.

For the 6WT, patients were given instructions on how to download the free 6WT smartphone app on their iOS or Android smartphone device (Maldaner et al., 2020a; Maldaner and Stienen, 2020). They were asked to self-measure their maximum walking ability over 6 ​min by walking as fast as possible on an even, relatively straight course (road or street outside), while the app would determine the 6-min walking distance (6WD) in meters (m) using global positioning system (GPS) coordinates. The reliability and accuracy of this app-based approach has been reported in previous studies (Maldaner et al., 2020a; Maldaner and Stienen, 2020; Stienen et al., 2019b).

### 2.3 Statistical & ethical considerations

Objective functional impairment (OFI) was calculated by the app, based on the patient’s sex and age, considering normal population data (Gautschi et al., 2016; Maldaner and Stienen, 2020; Tosic et al., 2020). The standardized 6WT results are expressed as Z-scores (standard deviations from the norm), according to the formula “Z-score = ((a – b)/c)”, where a ​= ​observed test time; b ​= ​normal population mean test time; c ​= ​normal population SD of test time. For the TUG test, Z-scores are transformed into T-scores, according to the formula “T-score ​= ​10 x Z-score + 100”. A T-score of 123 or lower is considered normal (Gautschi et al., 2016; Maldaner and Stienen, 2020).

We used descriptive statistics to correlate OFI to the physical condition of the patient perioperatively. Due to the limited sample size, no statistical tests were run. Change in function was evaluated based on published minimum clinically important difference (MCID) values, which are 2.1–3.4s for the TUG test and 92 ​m or a Z-score of 1.0 for the 6WT (Gautschi et al., 2017; Maldaner et al., 2021; Zeitlberger et al., 2021).

All patients signed the institutional waiver at hospital admission, consenting to non-identifiable use of their data for research purposes.

## 3 Results

### 3.1 Case 1: intradural, extramedullary tumor

A 51-year-old female presented as an emergency with a 3-month history of thoracolumbar pain with pseudoradicular pain radiating in her left abdomen and sensitive alterations in her right foot (paresthesia and numbness). In the 4 days prior to self-admission, she had experienced a progressive weakness in both legs with gait disturbances, including ataxia and decreased mobility.

**Fig. 1:**
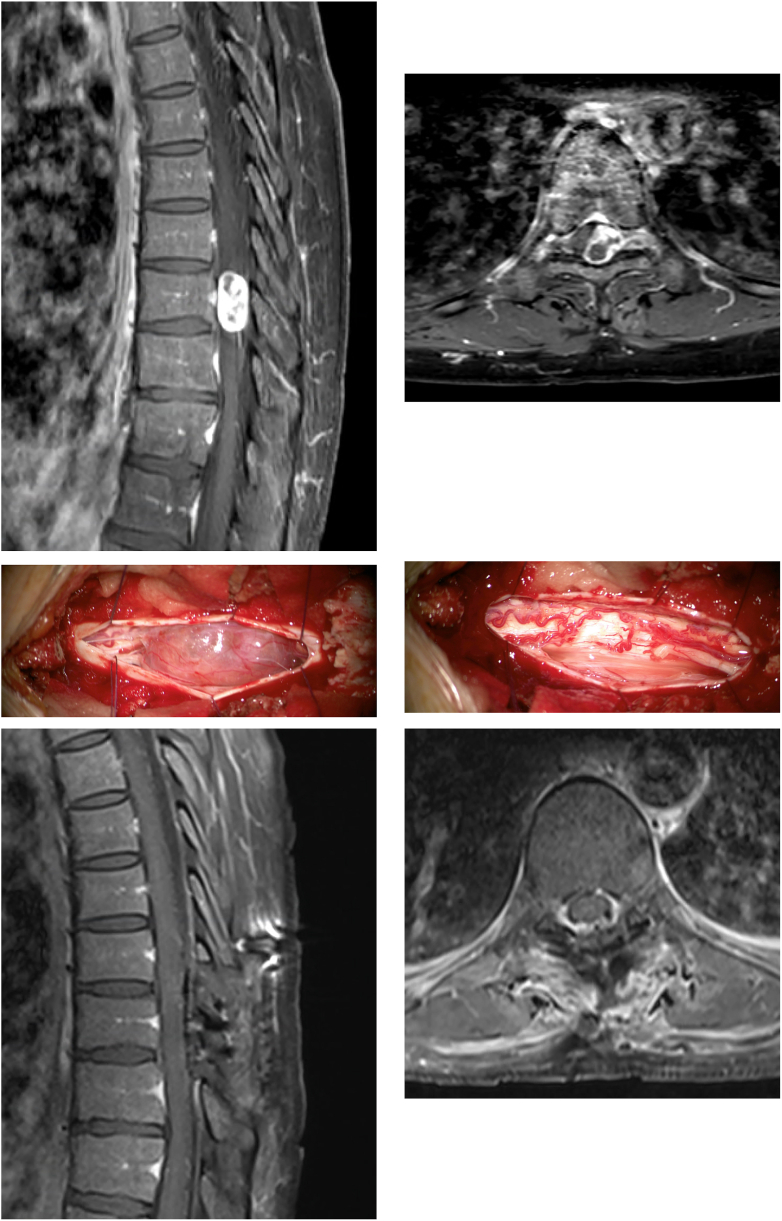
Case 1. **A:** Preoperative sagittal T1-weighted and contrast-enhanced MRI, revealing the round and partially cystic intradural, extramedullary lesion **B:** Axial view, illustrating extension into the left Th9 foramen **C:** Intraoperative view of the tumorous lesion after dural opening **D:** The tumor arose from the Th9 nerve root, which was transected to remove it completely Sagittal (**E**) and axial (**F**) T1-weighted and contrast-enhanced MRI on the first postoperative day demonstrates complete resection of the mass.

On examination, there was muscle wasting, paresis of both dorsiflexion and plantarflexion (4/5 on the BMRC scale), hyperreflexia of lower extremities, a bilateral Babinski sign, and a general hypoesthesia below Th10. The preoperative TUG-test revealed severe OFI with 33.6s and a corresponding age- and sex-adjusted T-Score of 269.4 (Fig. 1A). Magnetic resonance imaging (MRI) revealed a (1.4× 1.2 ​× ​2.4 ​cm) intradural, extramedullary partially cystic tumor, located at the level of Th9 with compression of the adjacent spinal cord but without evident T2-hyperintensity. Contrast-enhanced T1-sequences showed well-delineated enhancement of the lesion, extending somewhat into the left neuroforamen (Th9; Fig. 1B).

The patient underwent Th9 laminoplasty with microsurgical resection under electrophysiological monitoring on the admission day. After the durotomy, a firm grayish brown tumor was revealed with a good dissection plane (Fig. 1C). Total excision of the lesion (including the Th-9 fascicle) was possible (Fig. 1D), and was confirmed with intraoperative ultrasound. During the resection, we noticed improvement of somatosensory evoked potentials (SSEP) and motor-evoked potentials (MEP) of the lower left limb compared to baseline.

The patient had an uncomplicated postoperative recovery with initial persistence of the sensory deficits but immediate full recovery of the motor deficits. The postoperative MRI showed complete removal of the tumor (Fig. 1E–F). The atactic gait and overall mobility improved considerably until discharge on postoperative day seven. The TUG-test at discharge determined clinically meaningful improvement from severe to now moderate OFI with 13.4s and a T-score 145.4. Histopathological workup diagnosed a schwannoma (WHO Grad I).

After 3 weeks of in-patient rehabilitation, further slight improvements in her sensitivity (residual hypoesthesia along both legs) and the atactic gait was noted. At the 6-week outpatient clinic, the patient showed no residual OFI with a TUG test of 7.6s, corresponding to a T-score of 109.9. At three months postoperative the TUG test still revealed a walking function within the range of the normal population (6.8s, T-score of 104.9).

### 3.2 Case 2: ventral dural leak and spinal cord herniation

A 54-year-old female presented to our outpatient clinic with subacute right hemi-hypoesthesia below Th5 and intermittent paresis of the right leg that impaired her walking function when ambulating longer distances. The problems had begun with a severe impact to her back while bob sleighing in prone position 3 months previously. Initially the patient had experienced self-limiting thoracic and interscapular back pain including bilateral radiating chest pain.

**Fig. 2:**
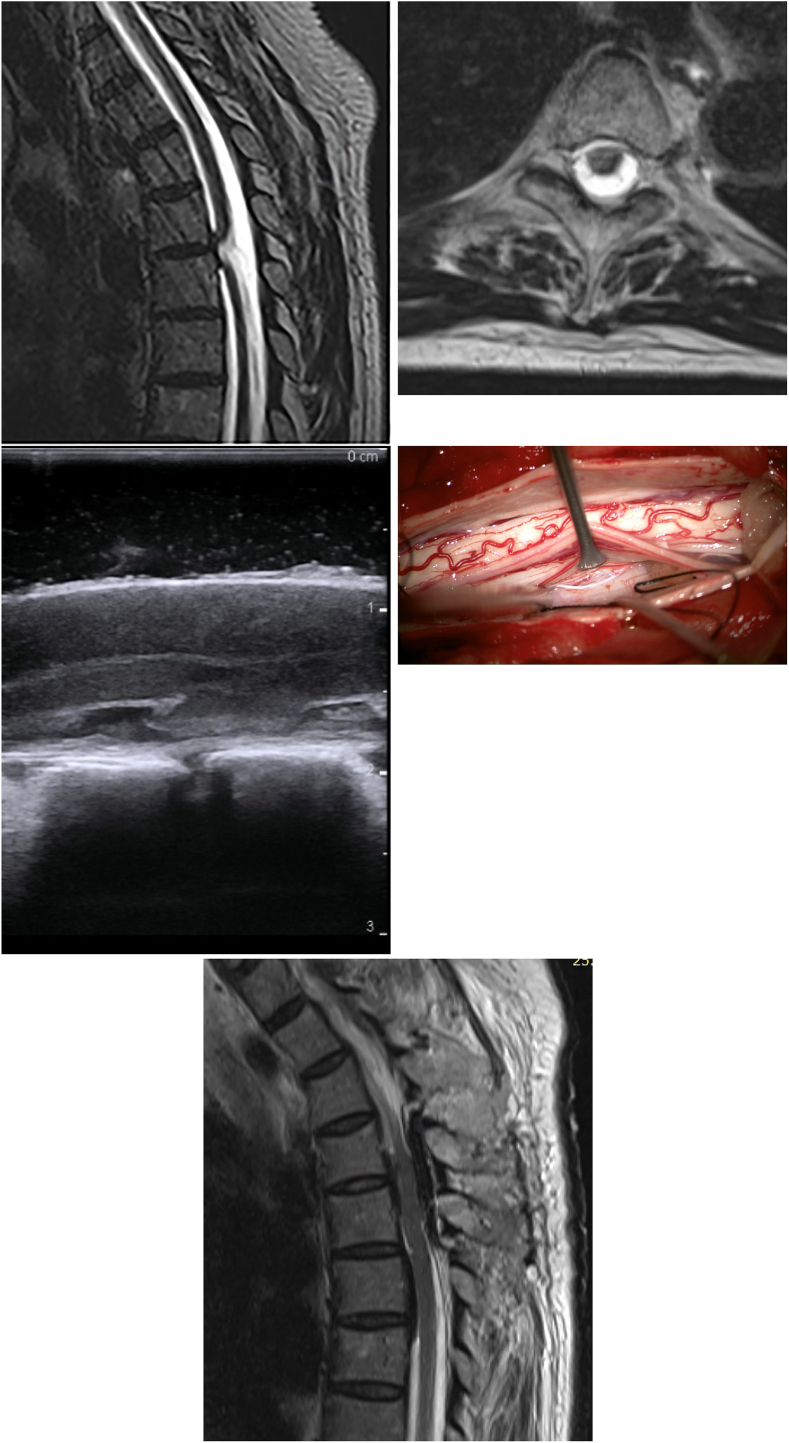
Case 2. **A:** Preoperative sagittal T2 (SPACE)-weighted MRI, demonstrating the ventral dural defect with spinal cord herniation at the height of the disc space Th4–Th5 **B:** Axial view representing left paramedian ventral spinal cord herniation and epidural fluid collection **C:** Intraoperative ultrasound examination displaying the ventral dural defect with spinal cord herniation before reduction **D:** Microscopic view on the dural defect before reduction **E:** Postoperative sagittal T2-weighted MRI showing minimal ventral myelopathy of the completely repositioned medulla spinalis, surrounded by pericardium patch.

On examination her motor functions were preserved, but she had a sensory deficit with numbness below Th5 to light touch on the right side of her body. Her baseline TUG test was 9.0s (T-score 118.6, no OFI). We asked her to self-determine the 6-min walking distance, which was also normal with 558 ​m and a Z-score of −0.09. Subsequent MRI studies showed a medial ventral dural defect with spinal cord herniation at the height of the disc space Th4–Th5 (Fig. 2A–B). After several weeks of observation with progression of symptoms, the patient decided to undergo surgery.

A Th4–Th5 laminoplasty was performed with MEP-, SSEP-, as well as D-wave monitoring. After a midline durotomy and release of the bilateral dentate ligaments, the spinal cord was gently rotated and the dural defect was exposed from both sides (Fig. 2C–D). Arachnoid adhesions were resolved and the dural defect was longitudinally extended with a diamond blade to allow for careful repositioning of the herniated spinal cord into the thecal sac. A bovine pericardium patch was placed ventrally in the “hammock technique” and sutured to the dura blades posteriorly before dural closure. Except for a short, temporary decrease in SSEPs with complete resolution until closure, the monitoring remained stable.

The patient reported improvement regarding her hypesthesia in the immediate postoperative period and gross motor function was preserved, but her gait stability seemed decreased and we requested in-patient rehabilitation. Postoperative MRI showed a minimal myelopathy of the ventral medulla spinalis (Fig. 2E). Her 6WD at 12 days postoperative (measured during rehab) was 466 ​m, corresponding to a clinically significant decline in function (Z-score of −1.2). About 1 month postoperatively the TUG-test revealed mild OFI (11s; T-score 130.7) and her 6WD was 470 ​m (Z-score −1.1). At the end of the rehab, she had no remaining OFI on the TUG-test (6s; T-score 100.0), which had improved from baseline by the MCID while on the 6WT (540 ​m; Z-score −0.3) no remaining OFI was noted. At the 2-month follow-up, her walking ability on the 6WT had increased to 595 ​m (Z-score 0.34), and was in the range of the preoperative assessment (difference less than the MCID).

### 3.3 Case 3: intramedullary tumor

A 52-year-old patient with familial Von-Hippel-Lindau disease and a known intramedullary hemangioblastoma at C4-5 was referred by our oncologists for neurosurgical consultation. The lesion had been diagnosed 12 years previously but had been stable and clinically silent. Over the past 3 months, however, the patient had experienced progressive weakness of the left leg and a gait disturbance.

**Fig. 3:**
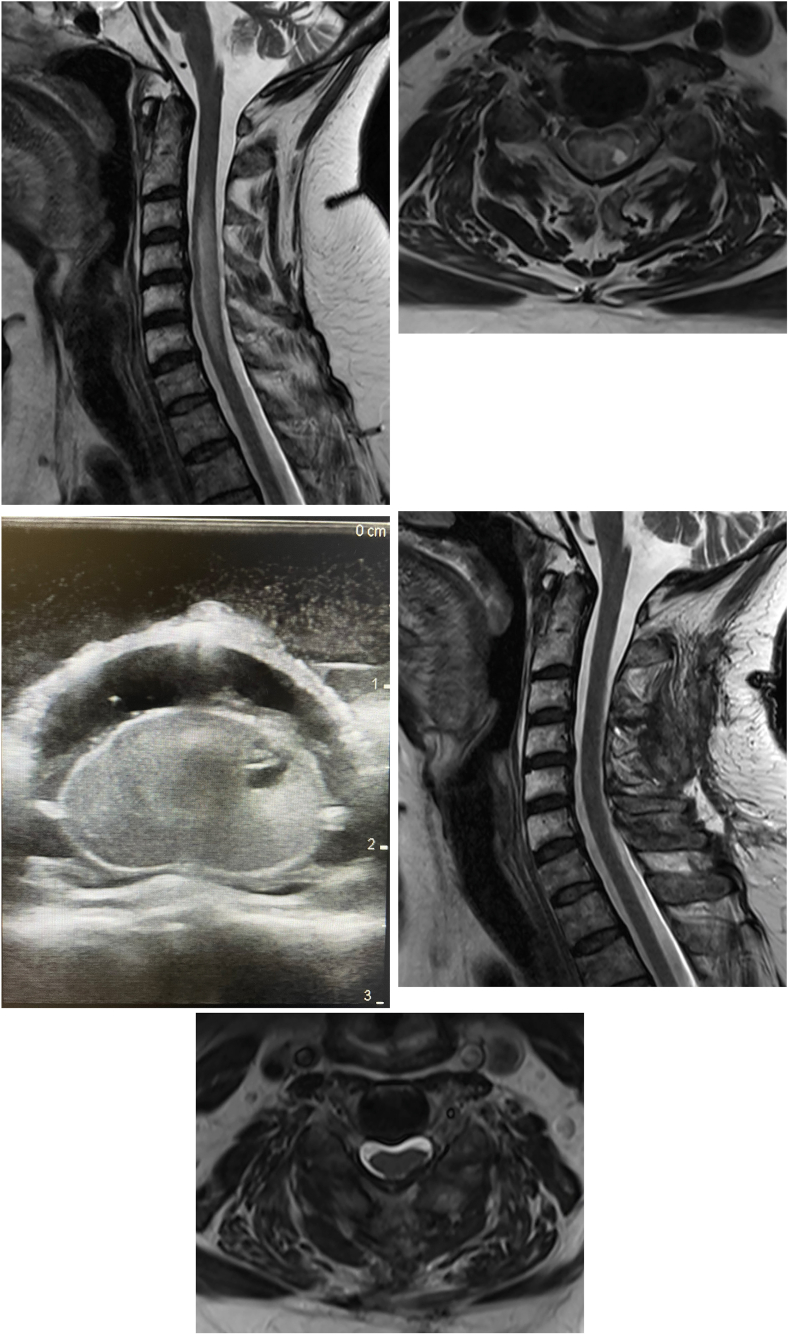
Case 3.**A:** Preoperative sagittal T2-weighted MRI, demonstrating extensive edematous intramedullary alterations from C2 to C7 **B:** Axial view at C4/C5, illustrating the intramedullary hemangioblastoma, originating in the posterolateral left column (0.4 ​× ​0.4 ​× ​0.2 ​cm) with a cystic part (0.4 ​cm in diameter) and adjacent edema **C:** Axial view using intraoperative ultrasound, demonstrating the intramedullary lesion **D:** Postoperative sagittal T2-weighted MRI, demonstrating complete resection and complete regression of peritumoral edema **E:** Axial view at C4/5 showing no residual tumor.

On examination the patient had a discrete left hemiparesis and hemiataxia with the lower extremity more affected than the upper extremity. She had hyperreflexia and bilateral cloni of the feet. Her gait was unsteady and atactic. She could walk fast on short distance (TUG test 9.6s, T-score 122.1) but her 6WD was severely limited (218 ​m; Z-score −4.1). MRI studies showed the known intramedullary lesion, situated in the posterolateral left column (0.4 ​× ​0.5 ​cm) at C4-5 with new, prominent spinal cord edema (Fig. 3A–B) and resection was recommended.

We performed C4 and C5 laminoplasty and microsurgical intralesional resection with ultrasound guidance under neurophysiological monitoring (SSEP, MEP & D-wave). The procedure was uneventful without reduction of potentials (Fig. 3C).

The postoperative examination revealed no new gross neurological deficits, but the patient reported a temporary deterioration of gait stability and slight ataxia. This dip in performance was substantiated with TUG-test evaluations two (18.7s; T-score 177.9; severe OFI) and seven days postoperative (14.4s; T-score 151.4; moderate OFI) that surpass the MCID. Postoperative MRI showed complete resection without any signs of complication (Fig. 3D–E). Histopathologic workup of the specimen confirmed the diagnosis of hemangioblastoma (WHO grade I). The patient was seen in the outpatient clinic six weeks postoperative, after completing rehabilitation. At this time she reported slight subjective improvement in her gait instability & ataxia compared to preoperative. On both the TUG-test (7.8s, T-score: 110.9) and the 6WT (269 ​m; Z-score −3.5) improvements from baseline were evident, but on both metrics they were less than the MCID.

### 3.4 Case 4: spinal cord tethering by arachnoid adhesions

A 62-year-old female presented with chronic right-sided spastic leg-, groin- and abdominal pain. Extensive medical assessments did not find any cause. Her patient history included aneurysmal subarachnoid haemorrhage with subsequent coiling of an intracranial aneurysm more than 10 years previously. In the preceding four years, she had developed progressive weakness of the right leg and an unsteady gait.

**Fig. 4:**
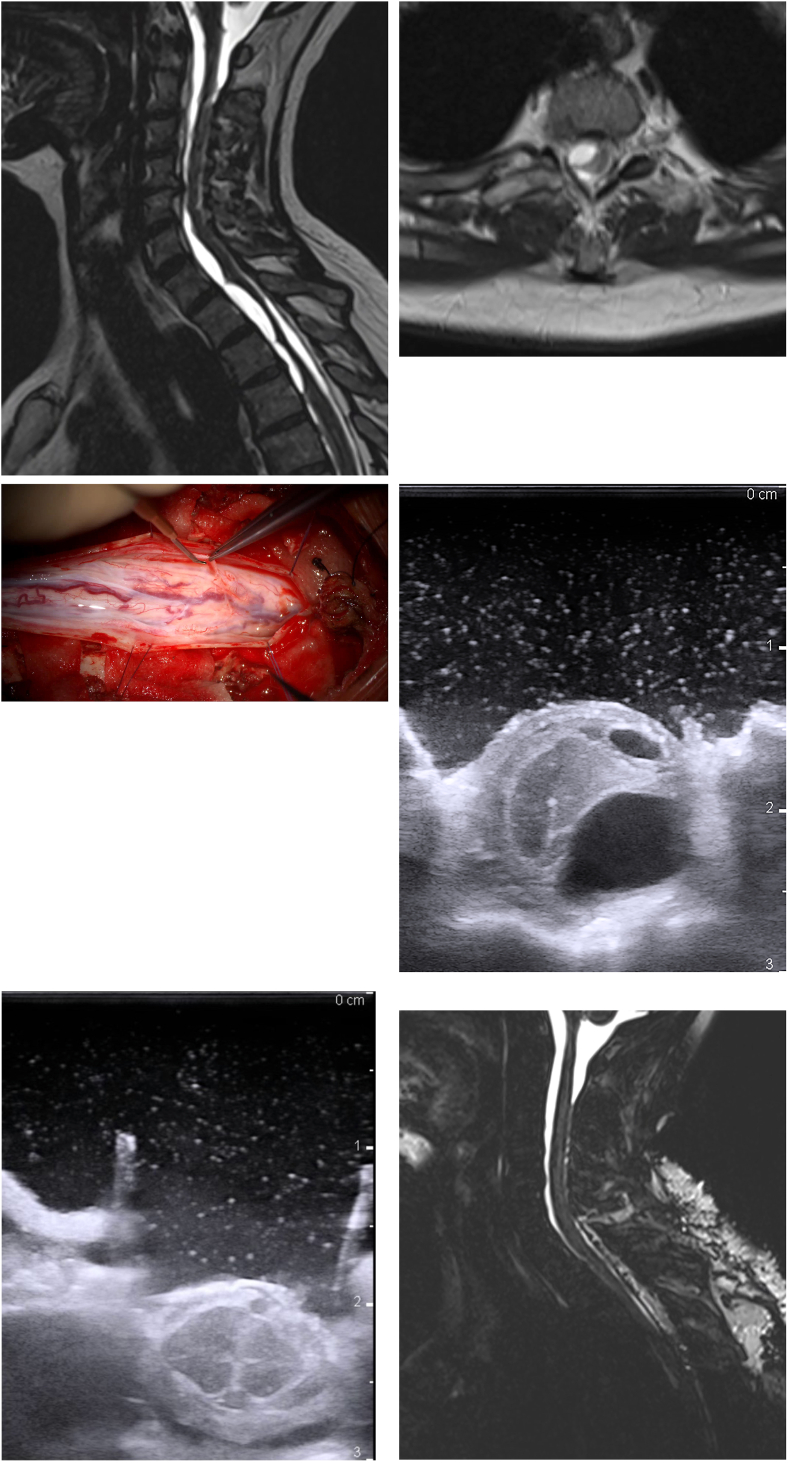
Case 4. **A:** Preoperative sagittal T2 (SPACE)-weighted MRI, illustrating multiple arachnoidal adhesions from C4 to Th8 and cystic arachnoid configurations leading to a significant spinal cord deconfiguration from C7 to Th2 **B:** Axial view at Th1/Th2, demonstrating right-sided T2-hyperintense cystic configurations displacing the spinal cord, causing myelopathy **C:** Intraoperative view after laminoplasty of C7–Th3 in “lobster-tail technique” and durotomy, revealing ubiquitous scarred arachnoidal adhesions **D:** Intraoperative ultrasound displaying arachnoid cyst formation compressing the spinal cord **E:** After adhesiolysis, cyst resection and extensive fenestration, no cystic formations were visible on intraoperative ultrasound **F:** The postoperative sagittal T2 (SPACE)-weighted MRI illustrates normalization of the spinal cord configuration and absence of adhesions and cyst formations.

Examination revealed Brown-Séquard syndrome with a discrete spastic paresis of the right leg and hyperreflexia. The depth perception, sense of position, temperature and pain of the right body half were impaired below Th5. Ambulation was unsteady and a right sided spastic gait-pattern was recorded. She scored 13.4s on the TUG-test (T-score 118.4). MRI studies showed multiple arachnoid adhesions between C4 – Th8, with right-sided cysts compressing the spinal cord mostly between C7 – Th3 and myelopathy at Th1-2 (Fig. 4A–B).

Cervico-thoracic laminoplasty of C7–Th3 was performed using the “lobster-tail technique”, followed by intradural exploration and microsurgical adhesiolysis under continuous neurophysiological monitoring (MEP, SSEP). The arachnoid cysts were identified and resected or extensively fenestrated until complete normalization of the spinal cord configuration was displayed on intraoperative ultrasound (Fig. 4C–D).

The patient experienced immediate improvement of the right sided limb weakness and spasticity. Postoperative MRI showed a regular spinal cord configuration in the operated spinal segments (Fig. 4E–F). After rehabilitation further improvement of gait stability and limb weakness was achieved. At the 6-week follow-up, she had a TUG-test of 8.8s (T-score 107.6; no OFI), representing clinically relevant improvement in function compared to preoperative.

## 4 Discussion

The purpose of this pilot study was to evaluate the feasibility of objective outcome measurement pre- and postoperatively for a variety of pathologies affecting the spinal cord. We included patients who underwent surgery as we wanted to study the pre- and postoperative clinical course by use of the objective outcome measures. It is the authors experience that most patients with intradural, extramedullary tumors improve immediately after surgery, while most patients - with diseases in which some degree of spinal cord manipulation cannot be avoided - experience a slight and temporary decline in function, with recovery over the course of some weeks. In the included patients – and as described in the case vignettes – this observation could be confirmed and first-time quantified with robust data. To the best of knowledge of the authors, standardized quantification of walking function is not yet routine in clinical care of most spine centers and this manuscript describes a simple and inexpensive way to implement this.

### 4.1 What are the benefits of objective outcome measures?

We included different pathologies, all of them showing clinical similarities, however. Pre- and postoperative neurological deficits with consecutive functional impairment may occur, which can be objectified using the 6WT and TUG test. Intradural lesions or malformations such as dural arterio-venous fistulas, arachnoid cysts, intra- or extramedullary tumors and also less frequent pathologies such as spinal cord herniation are usually treated surgically when they become symptomatic and/or demonstrate progression on imaging (Abd-El-Barr et al., 2018; Kotecha et al., 2019). Thus, patients may present with gait difficulties or neurological deficit, but deficits may be subtle and hard to diagnose. Moreover, the courses can vary considerably, as seen in our case vignettes where the patient of Case 1 made a progressive recovery by almost factor 5 (TUG test initially 33.6s and finally 6.8s), whereas other patients show a “rockier” course. The use of objective tests of function may facilitate the detection and accurate quantification of a patient’s longitudinal impairment (Stienen et al., 2019a; Bostelmann et al., 2016; Hartmann et al., 2016; Loske et al., 2018; Staartjes and Schroder, 2018), as exemplified in the case vignettes.

Raw test results can be used but their interpretation is difficult as most physicians are not aware of what test result represents a “normal finding” or when a test result should be considered “pathological”. Here, Z-scores and T-scores represent elegant ways to express impairment as a deviation from normal population data (Maldaner and Stienen, 2020). A 6WT Z-score of −4.1 as measured in the patient of Case 3 (see above) indicates that the patient’s test result is about 4 standard deviations (SDs) below the normal population mean and hence can be considered as severe disability. The patient of Case 2 (see above) had no detectable OFI before surgery but demonstrated a temporary decline in function of about 1.2 SDs of the normal population mean, before recovering and being 0.34 SDs above the preoperative value at 2 months postoperative.

The clinical significance of measured change in function can be interpreted, as MCID values have been described for both tests. In the patient of Case 1, for example, it is evident that immediately after surgery she had a considerable change in function, reflected by a change of about 20s from pre- (33.6s) to postoperative (13.4s). The MCID of the TUG test has been described to range between 2.1 and 3.4s for degenerative lumbar spine disease (Gautschi et al., 2017; Maldaner et al., 2021), which means that the patient is able to feel an improvement in function if the TUG test result changes by at least this amount. The same patient continued to improve on the TUG test by 5.8s until six weeks postoperative (7.6s), which again surpasses this MCID value. However, the further improvement between week six and week 12 (6.8s) was more subtle and below the MCID (0.8s), which the patient also felt as less significant further recovery (Gautschi et al., 2017; Maldaner et al., 2021). As another example, the patient in Case 3 improved in terms of 6WD from pre- (218 ​m; Z-score −4.1) to six weeks postoperative (269 ​m; Z-score −3.5) but this change in function was below the MCID of the 6WT (92 ​m; Z-score 1.0) (Zeitlberger et al., 2021), hence clinically insignificant and despite a feeling of subjective, slight improvement in walking function a clear benefit cannot be ascertained at this point. Further, longer-term measurements may be required in these situations to monitor progressive recovery following the intervention.

The longitudinal self-measurement of function allows the patient to self-monitor during the rehabilitation process, which empowers the patient to be an active partner and may even be helpful to detect complications early (e.g., when functional impairment does not improve but decline, such as in progressive myelopathy) (Maldaner et al., 2019, 2020b). It must be pointed out, however, that before these tests are validated for the use in a larger cohort of patients with spinal cord pathologies and disease-specific MCIDs and cut-offs have been determined, interpretation of test results is challenging. Until a larger validation study has provided more insight, we suggest implementing these functional tests to collect merely “exploratory” additional outcome data.

### 4.2 Strengths and limitations

We used well-established outcome measures from degenerative pathologies of the lumbar spine and applied them in a small but broad sample of pathologies that all lead to spinal cord dysfunction. Specifically, to the best of our knowledge, this is the first time the TUG test and the 6WT smartphone application have been used to quantify OFI in non-degenerative spine disease. The approach is innovative and currently, no standardized walking assessments are routine in the evaluation and aftercare of these patients. Two objective outcome measures were chosen that include typically impaired functions in patients with spinal cord affection, such as standing up, turning around, sitting down, and balancing while walking. Moreover, both tests are favorably considered by patients and test qualities are well studied (Stienen et al., 2019a; Gautschi et al., 2016; Maldaner et al., 2020a; Maldaner and Stienen, 2020).

The study is most of all limited by its small study population (n ​= ​4), which is why no further, sophisticated statistical analysis was possible. Moreover, no PROMs were available in these patients as most PROMs are not validated for these pathologies, which may have allowed us to better correlate the subjective with the objective functional status. Hence, the case vignettes remain descriptive for now, but future studies should incorporate further valid measures of impairment such as the modified Japanese Orthopedic Association (mJOA) score to cross-validate OFI in patients with spinal cord affection. Future studies should also determine which test is best in which pathology as more subtle forms of disability experienced by the patient were not always detected by the tests (Case 2).

## 5 Conclusion

The standardized objective measurement of functional impairment was feasible in this pilot study, which evaluated four patients with diseases affecting the spinal cord. OFI as determined by either the TUG test or the 6WT resembled the functional status of the patient. We were able to detect and quantify functional decline and/or improvement over time. A larger data collection is required to substantiate the broader use of objective outcome measures for these indications.

## Declaration of competing interest

The authors declare that they have no known competing financial interests or personal relationships that could have appeared to influence the work reported in this paper.

## Appendix

The “TUG app” is available free of charge in multiple languages at the Apple App Store (https://itunes.apple.com/de/app/tug-app/id1119087707?mt=8) and Google Play (https://play.google.com/store/apps/details?id=ch.webgearing.tugapp).

The “6WT app” is available in English, French, and German. It can be downloaded free of charge at the Apple App Store (https://apps.apple.com/ch/app/6wt-app/id1454002232) and Google Play (https://play.google.com/store/apps/details?id=ch.webgearing.walkingapp&amp;hl=en&amp;gl=US).
